# CdSe/ZnS Core-Shell-Type Quantum Dot Nanoparticles Disrupt the Cellular Homeostasis in Cellular Blood–Brain Barrier Models

**DOI:** 10.3390/ijms22031068

**Published:** 2021-01-22

**Authors:** Katarzyna Dominika Kania, Waldemar Wagner, Łukasz Pułaski

**Affiliations:** 1 Laboratory of Transcriptional Regulation, Institute for Medical Biology, PAS, Lodowa 106 Street, 93-232 Lodz, Poland; lpulaski@uni.lodz.pl; 2 Laboratory of Virology, Institute for Medical Biology, PAS, Lodowa 106 Street, 93-232 Lodz, Poland; 3 Laboratory of Cellular Immunology, Institute for Medical Biology, PAS, Lodowa 106 Street, 93-232 Lodz, Poland; wwagner@cbm.pan.pl; 4 Department of Molecular Biophysics, Faculty of Biology and Environmental Sciences, University of Lodz, Banacha 12/16, 90-237 Lodz, Poland

**Keywords:** nanoparticles, apoptosis, human brain endothelial cells, reactive oxygen species

## Abstract

Two immortalized brain microvascular endothelial cell lines (hCMEC/D3 and RBE4, of human and rat origin, respectively) were applied as an in vitro model of cellular elements of the blood–brain barrier in a nanotoxicological study. We evaluated the impact of CdSe/ZnS core-shell-type quantum dot nanoparticles on cellular homeostasis, using gold nanoparticles as a largely bioorthogonal control. While the investigated nanoparticles had surprisingly negligible acute cytotoxicity in the evaluated models, a multi-faceted study of barrier-related phenotypes and cell condition revealed a complex pattern of homeostasis disruption. Interestingly, some features of the paracellular barrier phenotype (transendothelial electrical resistance, tight junction protein gene expression) were improved by exposure to nanoparticles in a potential hormetic mechanism. However, mitochondrial potential and antioxidant defences largely collapsed under these conditions, paralleled by a strong pro-apoptotic shift in a significant proportion of cells (evidenced by apoptotic protein gene expression, chromosomal DNA fragmentation, and membrane phosphatidylserine exposure). Taken together, our results suggest a reactive oxygen species-mediated cellular mechanism of blood–brain barrier damage by quantum dots, which may be toxicologically significant in the face of increasing human exposure to this type of nanoparticles, both intended (in medical applications) and more often unintended (from consumer goods-derived environmental pollution).

## 1. Introduction

The blood–brain barrier (BBB) is a structural and functional entity with unique properties conferred by elements of microvasculature of the central nervous system (CNS). The BBB tightly regulates the movement of molecules, ions, and cells between the blood and the CNS [1]. The main structural components enabling this function are endothelial cells, pericytes, astrocytes, and microglia [2]. At the molecular level, tight junctions between endothelial cells and the strict regulation of transcellular transport pathways ensures the protection of the CNS against toxins, pathogens, pro-inflammatory mediators, and other potentially neurotoxic substances that could lead to injury [3]. Therefore, the potential to disrupt or damage the blood–brain barrier is an important toxicological feature of xenobiotics and externally introduced substances which has to be taken into account when evaluating their possible impact on human health. In some cases, physiological endogenous factors can lead to increased BBB permeability—e.g., astrocyte-derived matrix metalloproteinases [4]. Sometimes, such transient permeability window may be advantageous for therapeutic purposes in cases when the BBB restricts drug delivery to sites of pharmacodynamic action in the brain—e.g., in cancer or neurodegenerative diseases [5]. However, in most cases any injury to cellular elements of the BBB causes a deleterious disruption of barrier properties, exposing vulnerable neural tissue to a multitude of detrimental effects.

The explosive development of nanobiotechnology, mostly founded upon numerous classes of novel nanoparticles, has led to hope for innovative treatments and bioengineering tools, but the danger of toxic side effects from insufficiently studied products remains large [6]. It has been convincingly demonstrated for numerous nanoparticles that chemical and physical interactions with cellular components can lead to the unexpected disruption of important cellular functions [7]. Since many nanoparticles have the ability to interfere with cellular machinery crucial to the barrier phenotype in the BBB, such as cell–cell connections, endo-/exocytosis, and membrane transport, we decided to use well-established cellular models reproducing the most important features of the BBB in vitro to test the impact of exposure to typical nanoparticles. We selected two nanoparticle types which are widely held to exhibit divergent biocompatibility: quantum dots, which are nanometric semiconductor crystals containing heavy metal salts, for which the described toxic effects include metal ion leaching and electron transfer, leading to reactive oxidant formation [8]; colloidal gold nanoparticles, usually considered largely bioorthogonal [9]. While there exists a large body of published research on the mechanisms of nanoparticle transport through the BBB, BBB circumvention by nanoparticles for drug delivery purposes, and nanoparticle design for BBB crossing, there is a significant lack of mechanistic toxicological data on the actual impact of individual nanoparticle types on BBB function [10,11]. Our present study is a basic exploration of the molecular and cellular mechanisms of the potentially detrimental impact of nanoparticle exposure on features relevant to barrier efficiency in cultured brain microvascular endothelial cell lines, the cell type most directly involved in BBB function [12]. Our working hypothesis was that quantum dots would exert some sort of deleterious effect on the barrier cell phenotype, with gold nanoparticles serving as the expected negative control for this toxicity. Within the study, based on our initial results, we concentrated on the pro-apoptotic effects of nanoparticles, as these turned out to be most significant.

## 2. Results

### 2.1. Assessing Toxicity of Nanoparticles on hCMEC/D3 Cells

**Figure 1:**
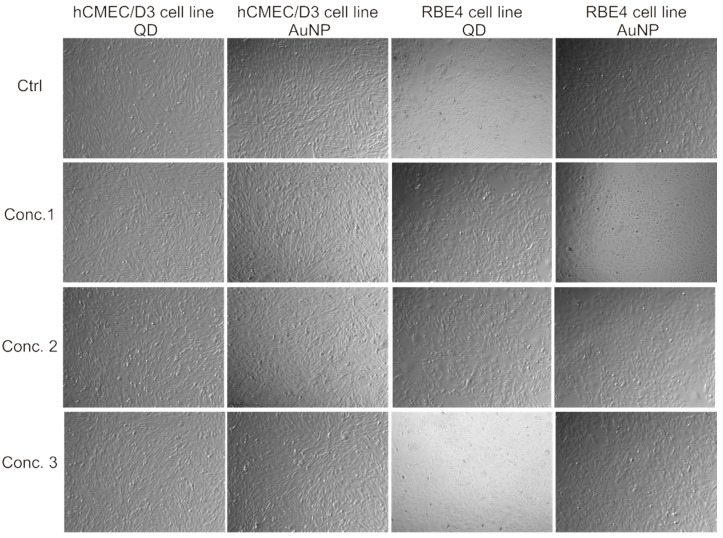
Morphology of brain microvascular endothelial cells (BBB cellular model) treated with nanoparticles. Microphotographs at 125× magnification taken using a Nikon Eclipse TE2000 microscope with a phase contrast objective. Columns one and two contain images of the hCMEC/D3 cell line, columns three and four of the RBE4 cell line. Concentrations of respective nanoparticles, labeled on the left and referring to each row of images, are as follows: for quantum dot nanoparticles (QD), concentration 1 is 0.1, 2–1, 3–10 µg/mL; for gold nanoparticles (AuNP), concentration 1 is 0.1, 2–0.5, 3–1 µg/mL.

We selected the well-established human microvascular endothelial cell line hCMEC/D3 as our primary cellular model of nanoparticle effect on BBB cells. In some experiments, we verified obtained results in parallel using the analogous rat cell line RBE4. We exposed these cells to quantum dots (QD), as nanoparticles with a potentially strong biological impact. For control purposes, we also performed experiments using well-characterized gold nanoparticles with minimal bioactivity. To verify these assumptions, we recorded the phenotype of in-vitro cultured monolayers of investigated cell line models after 24 h of nanoparticle treatment, seeing no apparent effect on cellular morphology even at relatively high concentrations (Figure 1).

**Figure 2:**
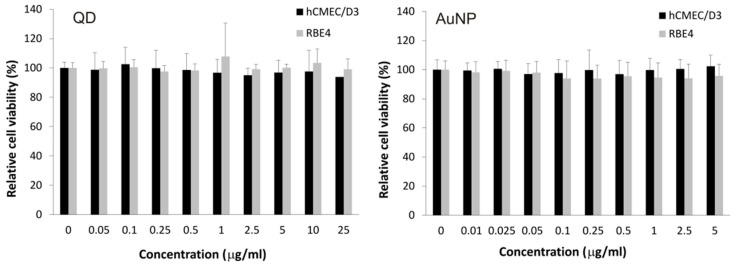
Survival of brain microvascular endothelial cell lines exposed to nanoparticles (QD and AuNP). Cell viability (for hCMEC/D3 and RBE4 cell lines) was assayed by the resazurin method, calculated as the rate of resazurin reduction by viable cells and presented as a percentage of the value for control (non-exposed) cells. Data shown as average ± S.D. (*n* = 6), no differences between depicted values were statistically significant at *p* < 0.05.

A cellular viability assay based on metabolic activity confirmed the lack of detectable toxic outcome in a broad concentration range for both tested nanoparticle types and cell lines after 24 h of treatment (Figure 2). This result was somewhat surprising in the case of quantum dots, so we pursued a deeper study of cellular functions to identify the putative expected biological effect of exposure to these particles.

### 2.2. The Impact of the Nanoparticles on Barrier Phenotype Properties in Brain Microvascular Endothelial Cells

**Figure 3:**
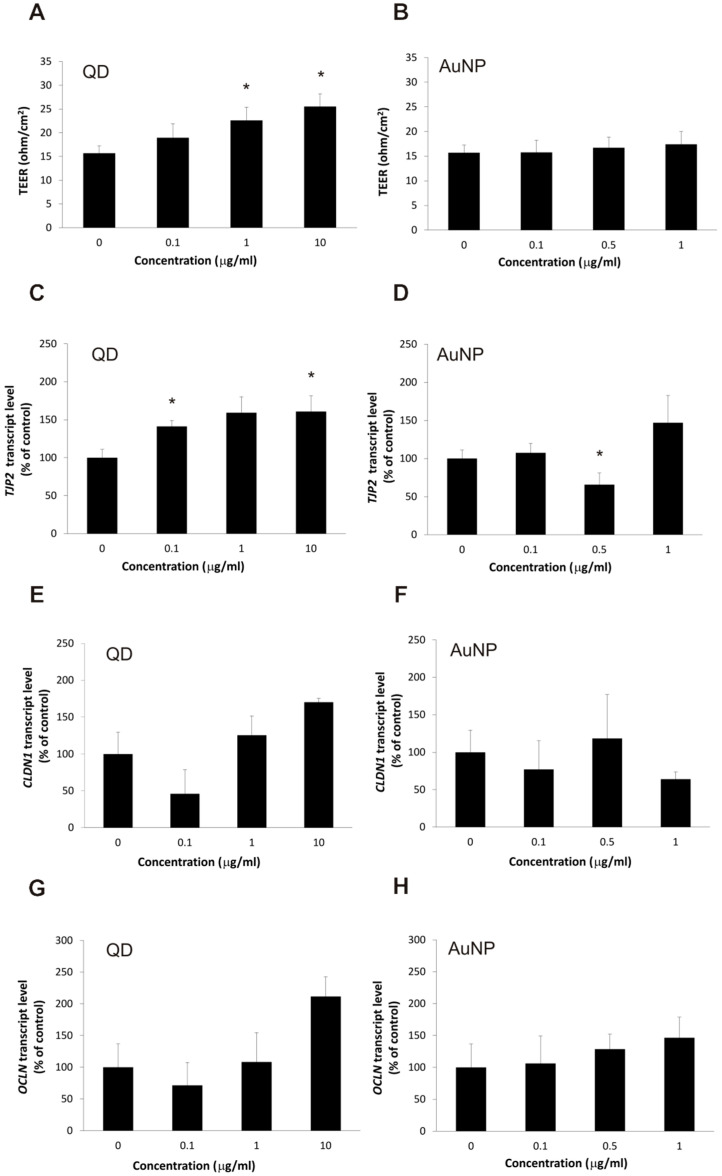
Barrier phenotype properties in brain microvascular endothelial cells exposed to nanoparticles. Paracellular transport barrier was assayed by measuring electrical resistance (TEER) in the hCMEC/D3 cell line treated with QD (panel (**A**)) and AuNP (panel (**B**)) nanoparticles. Calculated TEER values (reported TEER) measured using the Millicell multi-well volt-ohm meter. Data presented as average ± S.D. (*n* = 30), asterisks indicate differences from control (untreated cells) which were statistically significant at *p* < 0.05. Expression of tight junction proteins in the hCMEC/D3 cell line, treated with QD (panels (**C**,**E**,**G**)) and AuNP (panels (**D**,**F**,**H**)) nanoparticles, was assayed by real-time RT-PCR for cognate mRNAs (encoding ZO-2—panels (**C**,**D**); claudin 1—panels (**E**,**F**); and occludin—panels (**G**,**H**)), calculated as the relative copy number values per housekeeping gene index and presented as average ± S.D. (*n* = 3 in panels (**E**,**F**); *n* = 4 in panels (**C**,**D**,**G**,**H**)); asterisks indicate differences from the control (untreated cells) which were statistically significant at *p* < 0.05.

Since the barrier phenotype is a complex interplay of cellular functions, including cell–cell interactions and membrane transport, we decided to test the features of barrier phenotype at the in vitro level. The most commonly used parameter of capacity to prevent paracellular transport (BBB leakage), which is a marker of functional BBB integrity, is the ability to form a barrier for ionic current, measured as transendothelial electrical resistance (TEER). The results for hCMEC/D3 cells treated with nanoparticles for 24 h are presented in Figure 3A,B. While gold nanoparticles had no detectable impact on barrier function as expected, exposure to quantum dots, to our surprise, slightly increased the TEER value, indicating a possible decrease in the cell monolayer permeability to ions. Moreover, a threshold effect was observed with no significant difference between two highest applied QD concentrations, suggesting a mechanism related to cellular signaling rather than the physical blocking of ionic current.

The main cellular feature responsible for the paracellular transport barrier is the tight junction complex between neighboring endothelial cells. Our main cellular model, the hCMEC/D3 cell line, is known to exhibit plasticity of this complex at the molecular level, so we decided to test the impact of nanoparticles on the expression levels of genes coding for principal tight junction proteins. The results showed a significant increase in TJP2 gene expression (coding for the ZO-2 protein), while the increase was not significant for CLDN1 and OCLN (coding for claudin 1 and occludin, respectively), as shown in Figure 3C–H. No changes were seen for cells exposed to gold nanoparticles. While this is not a direct corroboration of the molecular mechanism of the observed TEER increase upon QD treatment, it provides a consistent model of short-term QD action through signaling pathways. On the other hand, the impact of nanoparticle exposure on genes encoding proteins important for other aspects of barrier function (membrane transporters, Wnt receptor elements) did not show any consistent pattern of modulation by either of the nanoparticle types (Supplementary Data, Figure S1).

### 2.3. Changes in Cellular Respiration and Redox Homeostasis Caused by Nanoparticles

**Figure 4:**
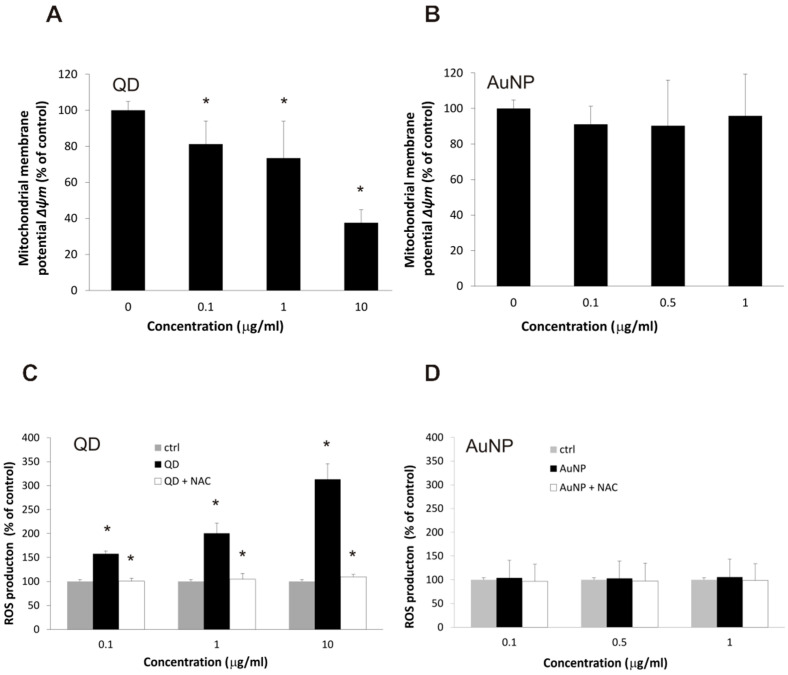
Cellular processes impacted by exposure to nanoparticles in brain microvascular endothelial cells. Mitochondrial potential (as a benchmark of cellular respiration) was measured by the JC-1 fluorescence method in the hCMEC/D3 cell line treated with QD (panel (**A**)) and AuNP (panel (**B**)) nanoparticles, calculated as the ratio of dimer to monomer fluorescence and presented as the percentage of value for control (non-exposed) cells. Data shown as average ± S.D. (*n* = 5), asterisks indicate differences from control (untreated cells) which were statistically significant at *p* < 0.05. Rate of reactive oxygen species production (as benchmark of redox homeostasis) was measured by the dichlorofluorescein fluorescence method in the hCMEC/D3 cell line treated with QD (panel (**C**)) and AuNP (panel (**D**)) nanoparticles with or without N-acetylcysteine (NAC) as a ROS scavenger, calculated as the rate of fluorescence increase and presented as the percentage of value for control (non-exposed) cells. Data shown as average ± S.D. (*n* = 8), asterisks indicate differences from control (untreated cells) which were statistically significant at *p* < 0.05.

Despite the apparent lack of deleterious effect of QD nanoparticles on the overall health phenotype of BBB cellular models, we decided to verify their effect on some intracellular processes (known from the literature to be targeted by quantum dots in other cellular models) and markers of their disruption. We selected two such processes—cellular respiration and redox homeostasis—verifying their integrity in hCMEC/D3 cells treated with quantum dots by measuring the mitochondrial membrane potential and the rate of reactive oxygen species generation, respectively. In this case, the experimental results conformed to expectations about the toxic potential of these particles (Figure 4). Even at the lowest applied concentration of 0.1 µg/mL, CdSe/ZnS nanoparticles depolarized the mitochondrial membrane (measured by the lipophilic indicator dye JC-1), reaching over 50% depletion at the highest tested concentration of 10 µg/mL, corresponding to a virtual collapse of the respiratory potential. Similarly, the intracellular production of reactive oxygen species (measured using the reactive fluorescent dye precursor dichlorofluorescin) increased in cells treated even with the lowest concentration of QDs, with a corresponding rise in this effect at higher concentrations. The specificity of the detection method was confirmed by the ability of N-acetylcysteine, a known reactive oxygen species scavenger, to reset the rate of ROS generation back to the basal value. This confirms the lack of direct H2DCF oxidation by QDs, since it could not be prevented by the scavenger. This also establishes the intracellular space as the location for ROS production, since the dye is introduced as an inert precursor and transformed to the oxidable indicator within the cell.

### 2.4. Apoptotic Changes in Brain Microvascular Endothelial Cells after Exposure to Nanoparticles

**Figure 5:**
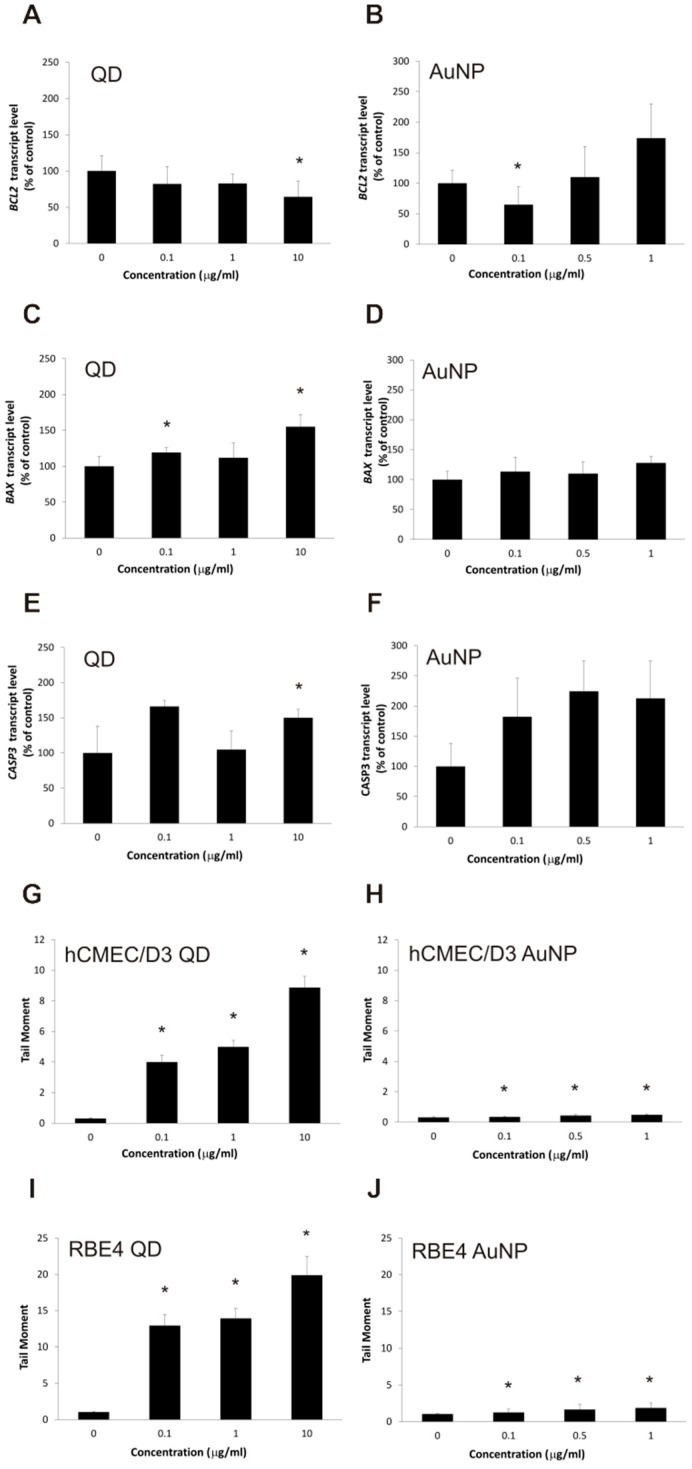
Apoptosis hallmarks in brain microvascular endothelial cell lines exposed to nanoparticles. Expression of apoptosis-related proteins in the hCMEC/D3 cell line, treated with QD (panels (**A**,**C**,**E**)) and AuNP (panels (**B**,**D**,**F**)) nanoparticles, was assayed by real-time RT-PCR for cognate mRNAs (encoding Bcl-2—panels (**A**,**B**); BAX—panels (**C**,**D**); and caspase 3—panels (**E**,**F**)), calculated as relative copy number values per housekeeping gene index and presented as average ± S.D. (*n* = 6 in panels (**C**,**D**); *n* = 8 in panels (**A**,**B**,**E**,**F**)). Asterisks indicate differences from control (untreated cells) which were statistically significant at *p* < 0.05. Nuclear DNA fragmentation in hCMEC/D3 (panels (**G**,**H**)) and RBE4 (panels (**I**,**J**)) cell lines, treated with QD (panels (**G**,**I**)) and AuNP (panels **H**,**J**) nanoparticles, was assayed by the alkaline comet method, calculated as the comet tail moment and presented as the average ± S.D. (*n* = 100). Asterisks indicate differences from control (untreated cells) which were statistically significant at *p* < 0.05.

Since we established the potential of QD treatment to disrupt some aspects of cellular homeostasis inside BBB cells, we set out to verify whether this may have a deleterious effect on cellular fate, such as programmed cell death. We measured the expression of genes encoding the most important apoptosis-regulating proteins: two genes from the Bcl family (BCL2 and BAX) and the key caspase (CASP3). The mRNA levels of all of these genes, measured by real-time RT-PCR assay, increased significantly after treatment with the highest concentration of QDs (10 µg/mL), as seen in Figure 5A–F. For lower concentrations, we did not observe a clear concentration dependence of effect, although the CASP3 expression was conspicuously stimulated by exposure to the lowest concentration (0.1 µg/mL). As these results hinted at a pro-apoptotic action of QD nanoparticles in BBB cells, we tested a commonly accepted hallmark of apoptosis, nuclear DNA fragmentation, using the alkaline comet assay to quantitate the potential effect. Figure 5G–J show a highly significant, conspicuous, concentration-dependent increase in comet tail moment value for QDs in both tested cell lines, with a much weaker, marginal effect for gold nanoparticles.

### 2.5. Nanoparticles Cause Changes in Plasma Membrane Asymmetry Related to Apoptosis

**Figure 6:**
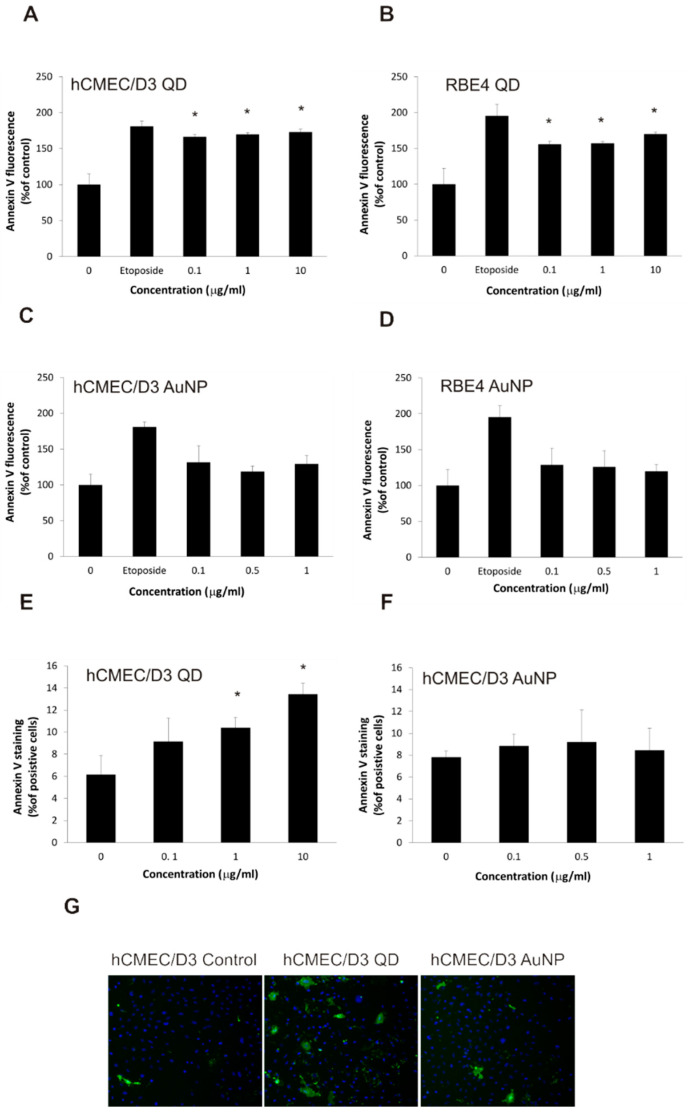
Phosphatidylserine exposure in brain microvascular endothelial cell lines exposed to nanoparticles. Annexin V binding to exposed phosphatidylserine in hCMEC/D3 (panels (**A**,**C**,**E**,**F**,**G**)) and RBE4 (panels (**B**,**D**)) cell lines, treated with QD (panels (**A**,**B**,**E**,**G**)) and AuNP (panels (**C**,**D**,**F**,**G**)) nanoparticles (as well as positive control etoposide), was assayed by bulk cell fluorescence using the EnVision plate reader (panels (**A**–**D**)) and high content screening using the ArrayScan VTi platform (panels (**E**–**G**)). Values for bulk cell fluorescence were calculated as the percentage of value for control (non-exposed) cells and presented as the average ± S.D. (*n* = 4 in panels (**A**,**B**), *n* = 3 in panels (**C**,**D**)). Asterisks indicate differences from control (untreated cells) which were statistically significant at *p* < 0.05. Values for high content screening were calculated as the percentage of positively stained cells and presented as the average ± S.D. (*n* = 4 in panel (**E**), *n* = 3 in panel (**F**)). Asterisks indicate differences from control (untreated cells) which were statistically significant at *p* < 0.05. Panel G shows representative images from the high-content screening platform.

In order to confirm the apoptotic mechanism of action of QDs on BBB model cell lines, we selected the highly specific early apoptosis hallmark of phosphatidylserine exposure in the external plasma membrane monolayer. Employing a standard binding assay for fluorescently labeled annexin V, we showed that QD treatment triggers apoptotic membrane asymmetry scrambling in both studied cell lines even at low concentrations (Figure 6A–D). The extent of this effect was somewhat weaker than for the control apoptotic toxin etoposide. The microplate-based phosphatidylserine exposure assay used in these experiments, based on the total amount of bound fluorescent label, has a threshold effect and is not well suited to demonstrating the concentration dependence of pro-apoptotic activity. Therefore, we applied a more advanced method based on a high-content screening analysis of annexin V binding at the level of individual cells (Figure 6E–G). This demonstrated unequivocally that quantum dots cause a dose-dependent apoptotic effect in hCMEC/D3 cells, with a more than double increase in the percentage of apoptotic cells after treatment with the highest concentration (10 µg/mL).

## 3. Discussion

The main reason for conducting the present study was the perceived imbalance in nanotoxicological studies on blood–brain barrier elements present in the literature. There is a wealth of studies on interactions between nanoparticles synthesized for medical purposes (drug delivery, antibiotics, or direct pharmacodynamic action) and the BBB with regard to the ability of these molecules to pass through the BBB or be excluded by it from the central nervous system—whichever property is more desired for the particular application [13,14,15]. Some of these studies include a toxicological element, where the direct impact of the nanoparticles on the BBB structural integrity and physiological condition is tested [16,17], but an overwhelming proportion of these publications still concerns nanoparticles which are designed for medical interventions in the human body, to be introduced into it in a planned and targeted manner. Still, increasing nanopollution and consequent unintentional exposure to environmental nanoparticles warrants enhanced research on all types of nanoparticles, especially with regard to their overall mechanisms of action at the level of cell biology and how they can impact the highly specialized function of the BBB. With this goal in mind, we decided to apply in vitro models of the BBB which have been long established both in the literature and in our laboratory [18,19,20,21] to evaluate the potential deleterious cellular events resulting from their exposure to model nanoparticles with previously described biological activity at this level. Therefore, the present study is mainly a generic exploration of nanotoxicological mechanisms at the cellular level as they apply to the BBB, rather than an application safety study for a specific medical application of the particular nanoparticles we studied.

Our main object of the studies was core-shell (cadmium selenide and zinc sulfide, respectively) quantum dot nanoparticles, synthesized and exploited industrially mainly due to their quantum state-related optical and electronic properties. Their physico-chemical characteristics which were most relevant to our study are their relatively small size (average diameter 6.2 nm), allowing for their potentially efficient cellular uptake both by triggered endocytosis and more passively by macropinocytosis [22,23]; poly-anionic carboxyl group surface modification, providing potential mechanisms of escape to cytoplasm [24]; and adsorptive interactions with important cellular components, including proteins [25] and mitochondrial lipids [26]; and the inclusion of two heavy metal elements in their structure, cadmium (in the core) and zinc (in the shell), which both have a plethora of known toxicological effects when in the form of free ions [27]. These QDs have a broad technological and biotechnological application range, extensively reviewed in [28], encompassing transistor and semiconductor nanoelectronics fabrication, production of lasers, solar cells and light-emitting diodes, medical imaging, and drug delivery.

Their capacity for toxic effects at the molecular and cellular levels has long been recognized, and they have been the subject of comprehensive toxicological studies. These investigations have revealed several mechanisms of toxicity, including intoxication by leached heavy metal ions, redox effects on the QD surface, and biomolecule adsorption [29,30,31]. This is worrying since, due to the increasing practical application of these particles in industry and in consumer goods, their presence in the environment begins to become a measurable element of nanopollution [27]. The complex toxicity pattern is, in this case, especially important, since the overall health burden may be influenced mainly by less overt, more devious mechanisms unrelated to direct cytotoxicity, which require research using specialized models relevant for individual tissues.

However, until now no direct determination of the impact of these important nanomaterials on the BBB either on the physiological (organ) level or in cellular models has been performed. For other nanoparticles, it is known that they may cause apoptosis of cells [32,33,34], damage mitochondria [35] or encourage prooxidant reactions [36,37]. For QDs, there are many toxicological studies on other types of cells in the CNS, including glial cells [38] and neuronal cells [39], in which toxic effects were demonstrated. More relevantly to our topic, since our cellular model are brain microvessel endothelial cells that form the central cell type of the BBB, studies on other endothelial cell types exposed to QDs were reported in the literature [32,40]. Some of the effects observed in these reports could be confirmed for BBB cells in our study (apoptosis, mitochondrial dysfunction, increased reactive oxygen species production), while others were more cell type-specific, similar to the tight junction effects observed here.

In our study, the specificity of the observed effects was demonstrated by the fact that gold nanoparticles of comparable size, expected to be taken up by the cells in similar ways to QDs [41] but, once inside, to remain largely bioorthogonal and without serious consequences to BBB-related cellular physiology [42], have been shown by us not to exhibit the effects that QDs had in our cellular models, including the pro-apoptotic activity as well as the interference with mitochondrial potential, redox homeostasis, and tight junctions. These findings lend weight to the previously suggested cellular mechanisms of toxicity, including interference with crucial protein activity and interactions as well as possible redox effects of exposed (uncoated) zinc sulfide (although direct reactive oxygen species generation has been excluded). Moreover, even correctly synthesized core-shell QDs are known to undergo the slow leaching of heavy metal ions in biological media and systems [30,31], and both zinc and cadmium have previously been shown to exert deleterious effects on BBB properties [43,44]. It is important to stress that BBB endothelial cells have been shown here to be relatively resistant to acute toxicity of QD nanoparticles, with no overt effects on viability of concentrations that killed bronchial epithelial cells [45] and fibroblasts [33] in other studies. Therefore, the toxic activity that we demonstrate for QDs is more devious, consisting of modifications to cellular function that are important for barrier activity, which both validates the cell-type-specific approach to nanoparticle biocompatibility testing and makes our findings highly relevant to further in vivo studies that would link the BBB disruption caused by QDs to the modulation of the pharmacokinetics and pharmacodynamics of other xenobiotic compounds which are—or are not—supposed to penetrate into the central nervous system.

While our study was initiated with the assumption (based on aforementioned and cited literature) that QDs would have profound negative effects on the health of brain microvessel endothelial cells, there were two elements to our findings that were unexpected within this paradigm: the relatively low intrinsic acute toxicity of investigated QDs and the fact that they seem to increase, rather than weaken, the ionic permeability barrier to transcellular transport (measured by TEER). In further studies, we furnish potential explanations for both of these initial findings. It seems that the investigated QDs exert their deleterious effects by an indirect mechanism with several steps, where apoptosis (mediated by an intrinsic pathway that involves the modulation of expression of mitochondrial apoptotic proteins and caspase 3) is accompanied by DNA damage, mitochondrial potential collapse, and reactive oxygen species production. All the three latter effects are probably secondary to the apoptosis process itself, being elements of the apoptotic chain of events rather than causatory phenomena. Permeability barrier disruption is a common result of treatment with some nanoparticles—e.g., anionic silica nanoparticles [46]—so our study adds an important novelty to BBB nanotoxicology in showing an opposite effect. In the context of these cellular events, ionic barrier enhancement is most probably a hormetic phenomenon stemming from the reactively activated expression of tight junction proteins which can be regulated by stress-related signalling pathways [47,48]. The existence of a similar phenomenon was previously demonstrated in a similar cellular model of brain microvascular endothelial cells exposed to lipopolysaccharide [49].

It is known that apoptosis in BBB cells is tightly regulated [50] and can be a clinically and physiologically very important mechanism of toxicity for long-term exposure to xenobiotics. Brain endothelial cell apoptosis may exacerbate the neurotoxicity of usually innocuous compounds, potentially endangering the brain with inflammatory infiltration. Our study, despite the expected intricacies of the blood–brain barrier physiology and studying BBB toxicology in a cellular model, can be interpreted as having confirmed the potential of QDs to exert a generally negative effect on CNS health. It is obvious that multi-parameter toxicological studies such as ours are highly necessary in this case, and we hope that this will find further development in the nanotoxicological community. The cellular model of the BBB, despite its limitations, demonstrated its effectiveness in identifying the underlying molecular mechanisms at the level of gene expression and cell biology. Of course, our tentative conclusions have to be confirmed in a more physiological in vivo model, which would be a part of a separate study.

## 4. Materials and Methods

### 4.1. Chemicals

The CdSe/ZnS quantum dots (SIGMA Aldrich, St. Louis, MO, USA) used in this study consisted of a nucleus composed of CdSe, protective layer composed of ZnS, and carboxyl groups on the surface. These nanoparticles had an average diameter of 6.2 nm. Gold nanoparticles (SIGMA Aldrich, St. Louis, MO, USA) were uncoated, with average diameter of 5 nm, delivered as a colloidal suspension in PBS.

Endothelial cell basal medium 2 (EBM-2) (Lonza, Lievres, Belgium), fetal bovine serum Superior (FBS) (Biochrom, MERCK), Pen-Strep, Chemically Defined Lipid Concentrate, HEPES, resazurin sodium salt (7-Hydroxy-3H-phenoxazin-3-one-10-oxide sodium salt) trypsin-EDTA, HBSS obtained from Gibco, Thermo Fisher Scientific (Waltham, MA, USA), Dulbecco’s phosphate-buffered saline, minimum essential medium Eagle (MEM), Ham’s F10 medium, L-ascorbic acid, fibronectin, rat collagen I, Hoechst 33342, hydrocortisone, human basic fibroblast growth factor (bFGF), gold nanoparticles, CdSe/ZnS quantum dots, N-Acetyl-L-cysteine (NAC), TRI reagent, 2-propanol, chloroform were purchased from SIGMA Aldrich (USA), recombinant human Fibroblast Growth Factor basic, LPS, TNFα—R&D Systems, Inc., Oakville, ON, Canada). Anexinn V Alexa Fluor^®^ 488 and H2DCF-DA were obtained from Life Technologies (Renfrew, UK). Caspase-Glo^®^ 3/7 Assay Systems, JC-1 Dye and Maxima First Strand cDNA Synthesis Kit for RT-qPCR (Promega), was obtained from Thermo Scientific/Fermentas (Vilnius, Lithuania), whereas primers for PCR reaction were from (Oligo.pl, Warsaw, Poland) and SYBR Green I Master Mix were purchased from Roche (Basel, Switzerland). The other chemicals were purchased from POCH S.A. (Gliwice, Poland) if not otherwise indicated and were of the highest available analytical purity. Tissue culture flasks and plates were from (PAA, München, Germany).

### 4.2. Cell Culture

The in vitro studies were carried out on the human immortalized brain endothelial cell line (hCMEC/D3) (kindly donated by Prof. Pierre Couraud from INSERM, Paris, France). These cells were originally derived from isolated human microvascular brain endothelial cells transformed with hTERT and with the Simian virus 40 antigen [18]. hCMEC/D3 cells have properties characteristic for the blood–brain barrier including expression of functional ABC transporters ABCB1, ABC3, and ABCG2. The cells were seeded onto culture flask previously coated with collagen I (150 µg/mL, at 37 °C, for the least one hour) and maintained in endothelial cell basal medium 2 (EBM-2) (Lonza, Lievres, Belgium), containing 5% FBS (fetal bovine serum), 1% pen-strep, hydrocortisone (1.4 µM), ascorbic acid (5 µg/mL), 1% chemically defined lipid concentrate, HEPES (10 mM), bFGF (1 ng/mL) in standard conditions: 37 °C, 100% humidity and the atmosphere being 5% CO_2_ and 95% air. Experiments were performed on cells from the passages between 26 and 35. Additionally, we also used rat brain endothelial cell line RBE4 in some experiments. Primary cultures of RBE cells were transformed with adenovirus E1A and selected on the basis of endothelial morphology and the expression of endothelial markers [51]. RBE4 cells were grown in MEM/Ham’s (1:1), supplemented with 10% FBS, 1% pen-strep and bFGF (1 ng/mL). The cells were cultured in cultured flasks pre-coated with collagen. Both cell lines were periodically tested for Mycoplasma, using MycoAlertTM Mycoplasma Detection Kit (Lonza, Lievres, Belgium).

### 4.3. Cell Treatment

Cells at a density suitable for the performed assay were incubated in a monolayer and culture conditions with nanoparticles or nanoparticles and other compounds for 24 h and then used for further analyses. All solutions of nanoparticles were prepared in the cultured medium, quantum dots were suspended and colloidally dispersed by sonication (30 min. at RT). This dispersion allowed the investigated nanoparticles to remain in suspension (without sedimentation) for the duration of the experiment. Control cells were treated with a corresponding volume of medium (without nanoparticles) according to the same schedule.

### 4.4. Cytotoxicity Assay

Cells (1 × 10^5^) in 0.1 mL culture medium per well were seeded into black flat-bottomed 96-well microtiter plates (PAA, Germany). The cells were exposed to different concentrations of nanoparticles for 24 h (5% CO_2_, 37 °C, 100% humidity) and cell viability was estimated by resazurin assay. At the end of incubation time medium was carefully removed and a monolayer of cells was washed with PBS and 0.l ml of resazurin solution (0.0125 mg/mL in HBSS) was added to each well. The plates were incubated at 37 °C. After 2 h incubation, the fluorescence intensity of resorufin was measured at λ_Ex_ = 380 nm and λ_Em_ = 500 nm, with a microplate reader (EnVision^®^ Multilabel Reader, Perkin Elmer, Waltham, MO, USA) The percentage of viable cells was calculated by comparing the mean value of fluorescence in the compound-treated cells to control, non-treated cells.

### 4.5. TEER Measurement

For the TEER (transendothelial electrical resistance) measurement, we used the transwell inserts (ThinCertTM, Greiner Bio-One, Frickenhausen, Germany). The hCMEC/D3 and RBE4 cell lines were plated onto sterile 24-well cell culture inserts on the top side, at 25,000 cells/cm^2^ (pore diameter 0.4), coated with collagen I and grown to confluence approximately five days. Before treatment with the nanoparticles, TEER was measured five times in every insert, using the epithelial volt-ohm meter Millicell^®^ ERS-2 (Millipore, Molsheim, France) with MERSSTX01 electrode. Wells showing aberrantly low TEER values were eliminated from the measurements. After treatment, the measurement was made again in the same wells. All TEER values were determined after subtracting the background (TEER for cell-free insert coated with collagen I) and by correction for surface area. The values were >40 Ω cm^−2^ [52].

TEER _REPORTED_ = (TEER _TOTAL_ − TEER _BLANK_) × M _AREA_.

### 4.6. Quantitative Real-Time RT-PCR

Total cellular RNA was isolated from a confluent monolayer of hCMEC/D3 cells incubated with the analyzed nanoparticles for 24 h, using TRI Reagent from Sigma (St. Louis, MO, USA), following the manufacturer’s instructions. The concentration and purity of the RNA were determined using a NanoDrop 2000 UV–Vis Spectrophotometer (Thermo Fisher Scientific, Waltham, MA, USA). The RNA was then reverse-transcribed with the Maxima First Strand cDNA Synthesis Kit for RT-qPCR (Thermo Scientific/Fermentas, Vilnius, Lithuania). Real-time RT-PCR was performed with SYBR-green PCR master mix (Roche, Basel, Switzerland) in a real-time PCR machine, LightCycler 480 (Roche). The primer sequences are listed in Supplementary data (Table S1) (*TJP2*, *CLDN1*, *OCLN*, *DKK1*, *DKK3*, *LRP5*, and *LRP6BCL2*, *BAX*, *CASP3*). qRT-PCR was performed at 94 °C for 4 min, followed by 40 cycles at 94 °C for 15 s, at 60 °C for 25 s, and at 72 °C for 25 s. Duplicate technical measurements of Ct were used to determine the consistency of amplification and averaged to obtain a single biological replicate. Quadruplicate biological measurements were used to detect the expression of target gene with normalization to the housekeeping genes used as an internal control—hydroxymethylbilane synthase (HMBS) and hypoxanthine phosphoribosyltransferase 1 (HPRT1). For data presentation, the ΔCt values were transformed into relative copy number values, the number of mRNA copies of the examined genes per housekeeping gene index, calculated as the average Ct value of the HPRT1 and HMBS housekeeping genes [53,54].

### 4.7. Estimation of Mitochondrial Membrane Potential (Δψm)

hCMED/D3 and RBE4 cells were seeded into 96-well microplates at a density 1 × 10^4^/well. After 24 h, QDs and AuNPs in three investigated concentrations were added to each well. The cells were incubated with the analyzed compounds for 24 h. At the end of the treatment time, the medium was removed and the cells were incubated in darkness with 5 µM JC-1 in HBSS for 30 min at 37 °C. The fluorescence of both JC-1 monomers and dimers was measured on a microplate reader (EnVision^®^ Multilabel Reader, Perkin Elmer, Waltham, MO, USA), using filter pairs of 530 nm/590 nm (dimers) and 485 nm/538 nm (monomers), [55]. We monitored the ratio of dimer to monomer of JC-1 in relation to the control, untreated cells.

### 4.8. Determination of Reactive Oxygen Species Production

The intracellular ROS production was recorded by monitoring changes in the fluorescent probe—2′7′-dichlorodihydrofluorescein diacetate (H2DCF-DA). The hCMEC/D3 cells were plated onto 96-well black plates (1 × 10^5^ cells/well) for 24. After that, the cells were incubated for 24 h with the nanoparticles under culture conditions. After treatment with nanoparticles, the cells were incubated with 5 µM H2DCF-DA for 30 min at 37 °C, in HBSS. Fluorescence intensity was monitored with a microplate reader (EnVision^®^ Multilabel Reader, Perkin Elmer, Waltham, MO, USA), at λEx = 492 nm and λEm = 517 nm. In some experiments NAC (3 µM) was added, 2 h before treatment with QDs.

### 4.9. Comet Assay

To estimate the genotoxic effect of the investigated nanoparticles, we used single-cell gel electrophoresis (SCGE) in the form of alkaline comet assay according to the procedure of [56]. In our experiments we applied the Comet Assay Kit (Trevigen, Minneapolis, MN, USA). The cells were plated onto a 24-well transparent plate. After 24 h the cells were treated with nanoparticles for a further 24 h. After this time, the cells were trypsinized and suspended in low melting point agarose in PBS, pH 7.4. 50 mL of cell suspension was spread on microscope slides supplied in Comet Assay Kit. After gelling, the slides were treated with lysis buffer containing 2.5 M NaCl, 100 mM EDTA, 1% Triton X-100, 10% DMSO and 10 mM Tris, pH 10 at 4 °C for 1 h. Slides were then placed in the electrophoresis solution (300 mM NaOH, 1 mM EDTA, pH > 13) for 40 min. Electrophoresis was carried out at 0.73 V/cm, 300 mA for 30 min. The slides were then neutralized, stained with 2 mg/mL DAPI and analyzed under fluorescent microscope (Nikon Eclipse TE2000, Nikon, Tokyo, Japan). All the steps of this procedure were performed in the dark. The level of DNA damage was determined on the basis of the comet tail moment using CaspLab software (1.2.3beta1 version, Comet Assay Software Project, Wrocław, Poland) [57,58].

### 4.10. Annexin V Binding Assay

Early apoptosis in nanoparticles treated cells was measured using Annexin V binding assay. the cells were seeded at the density of 3.5 × 10^3^ cells/well on a 96-well plate and treated with quantum dots or gold nanoparticles. Following 48 h incubation hCMEC/D3 cells were processed for Annexin V staining as described earlier by Wagner et al. Briefly, cells were stained with 10 μg/mL Hoechst 33,342 for 20 min, washed with staining buffer (10 mM HEPES, 140 mM NaCl, 2.5 mM CaCl_2_, pH 7.4), and incubated with Annexin V conjugated with Alexa Fluor 488 (Life Technologies) for 20 min at RT in dark. The plate was analysed using an ArrayScan VTI HCS Reader equipped with a 10× objective. Images of 16 fields per well were acquired, and cellular fluorescence intensity was analyzed using Cell Health Profiling Bioapplication V3 software (Cellomics, Waltham, MA, USA). Experiments were performed three times, each in six replicates. The data are presented as the mean ± S.D. of the percentage of positively stained cells, exhibiting greater average cellular fluorescence than the threshold calculated based on the untreated population [59].

### 4.11. Statistical Analysis

Data are expressed as means ± S.D. Statistical comparisons were evaluated using one-way ANOVAs and Bonferroni’s *t*-test.

## 5. Conclusions

In our study, we found that CdSe/ZnS quantum dots are not acutely toxic to hCMEC/D3 and RBE4 cells (human and rat in vitro models of the blood–brain barrier), but exert a pronounced pro-apoptotic effect accompanied by a collapse of mitochondrial membrane potential and antioxidant homeostasis. On the other hand, paracellular barrier properties are slightly improved, accompanied by the induction of tight junction protein genes. None of these effects were observed for bioorthogonal gold nanoparticles applied as a control. This complex bioactivity of nanoparticles that are increasingly common in the environment underscores the need for multi-parametric nanotoxicological studies in specialized models of tissue function.

## Data Availability

The data presented in this study are available on request from the corresponding author.

## Supplementary Materials

The following are available online at https://www.mdpi.com/1422-0067/22/3/1068/s1: Figure S1: Wnt signaling pathway in brain microvascular endothelial cell lines exposed to nanoparticles. Expression of Wnt-related proteins in hCMEC/D3 cell line, treated with QD (panels A, C, E, and G) and AuNP (panels B, D, F, and H) nanoparticles, was assayed by real-time RT-PCR for cognate mRNAs (encoding Dickkopf1—panels A and B, Dickkopf3—panels C and D, LRP5—panels E and F, and LRP6—panels G and H), calculated as relative copy number values per housekeeping gene index and presented as average ± S.D. (*n* = 4). No differences between the depicted values were statistically significant at *p* < 0.05.

## Abbreviation

BAX	BCL2 Associated X
BCL2	B-cell lymphoma 2
DKK1	Dickkopf-related protein 1
DKK3	Dickkopf-related protein 3
DMSO	Dimethyl Sulfoxide
EDTA	Ethylenediaminetetraacetic acid
HBSS	Hank’s balanced salt solution
hTERT	Human telomerase reverse transcriptase
LRP5	LDL Receptor Related Protein 5
LRP6	LDL Receptor Related Protein 6
MEM	Minimum Essential Medium
PBS	Phosphate buffered saline
TJP2	Tight Junction Protein 2
