# Supplementary material for: CdSe/ZnS Core-Shell-Type Quantum Dot Nanoparticles Disrupt the Cellular Homeostasis in Cellular Blood–Brain Barrier Models
Source: Int J Mol Sci. 2021 Jan 22;22(3):1068. doi: 10.3390/ijms22031068 (PMC7866238; doi:10.3390/ijms22031068)
Supplement: Supplementary file 1 [file ijms-22-01068-s001.zip › Supp. Data_ZIP/Table S1_primer sequenceKDKania_IJMS.doc]

| Table S1 Sequences of the primers used for real-time PCR | | |
| --- | --- | --- |
| **Name** | **Strand** | **Sequence** |
| *HPRT1* | Forward | TGACACTGGCAAAACAATGCA |
|  | Reverse | GGTCCTTTTCACCAGCAAGCT |
| *HMBS* | Forward | TGACACTGGCAAAACAATGCA |
|  | Reverse | GGGTACCCACGCGAATCAC |
| *TJP2* | Forward | TGTGAGGATTGGGAACGAGTTGG |
|  | Reverse | CTGGCCATTTGTTCAGCTCTGC |
| *CLDN1* | Forward | CCGTTGGCATGAAGTGTATG |
|  | Reverse | AAGGCAGAGAGAAGCAGCAG |
| *OCLN* | Forward | TCCATTGGCAAAGTGAATGA |
|  | Reverse | AGAGGTGCTCTTTTTGAAGG |
| *CASP3* | Forward | CGAAATTCAAAGGATGGGCTCCTGGTT |
|  | Reverse | CGGTTAACCCGGGTAAGAAATGTGCAT |
| *BAX* | Forward | TGCCTCAGGATGCGTCCACCAA |
|  | Reverse | CGGCAATCATCCTCTGCATGCTCCAT |
| *BCL2* | Forward | GCACGCTGGGAGAAAGGGTACGAT |
|  | Reverse | CACATCTCCAGCATCCCACTCGTA |
| *DKK1* | Forward | TTGACAACTACCAGCCGTACCC |
|  | Reverse | TGGGACTAGCGCAGTACTCATC |
| *DKK3* | Forward | AAGGAGCCACGAGTGCATCATC |
|  | Reverse | AGGTGTACTGGAAGCTGGCAAAC |
| *LRP5* | Forward | ACCGTACAGGCCCTACATCATTCG |
|  | Reverse | TGTAGTCGCTGTCACACACGTC |
| *LRP6* | Forward | CTGACAACAGGACTTGTAGTGCTC |
|  | Reverse | CAATCACCATGCGGTTGATGGC |
